# The Relationship between the Spatial Arrangement of Pigments and Exciton Transition Moments in Photosynthetic Light-Harvesting Complexes

**DOI:** 10.3390/ijms221810031

**Published:** 2021-09-17

**Authors:** Roman Y. Pishchalnikov, Denis D. Chesalin, Andrei P. Razjivin

**Affiliations:** 1 Prokhorov General Physics Institute of the Russian Academy of Sciences, 119991 Moscow, Russia; genoa-and-pittsburgh@mail.ru; 2 Belozersky Research Institute of Physico-Chemical Biology, Moscow State University, 119992 Moscow, Russia; razjivin@belozersky.msu.ru

**Keywords:** purple bacteria, bacteriochlorophyll, exciton theory, LH2 complex, LH1 complex, exciton transition moments

## Abstract

Considering bacteriochlorophyll molecules embedded in the protein matrix of the light-harvesting complexes of purple bacteria (known as LH2 and LH1-RC) as examples of systems of interacting pigment molecules, we investigated the relationship between the spatial arrangement of the pigments and their exciton transition moments. Based on the recently reported crystal structures of LH2 and LH1-RC and the outcomes of previous theoretical studies, as well as adopting the Frenkel exciton Hamiltonian for two-level molecules, we performed visualizations of the LH2 and LH1 exciton transition moments. To make the electron transition moments in the exciton representation invariant with respect to the position of the system in space, a system of pigments must be translated to the center of mass before starting the calculations. As a result, the visualization of the transition moments for LH2 provided the following pattern: two strong transitions were outside of LH2 and the other two were perpendicular and at the center of LH2. The antenna of LH1-RC was characterized as having the same location of the strongest moments in the center of the complex, exactly as in the B850 ring, which actually coincides with the RC. Considering LH2 and LH1 as supermolecules, each of which has excitation energies and corresponding transition moments, we propose that the outer transitions of LH2 can be important for inter-complex energy exchange, while the inner transitions keep the energy in the complex; moreover, in the case of LH1, the inner transitions increased the rate of antenna-to-RC energy transfer.

## 1. Introduction

Taking into account the current number of studies [1] devoted to the comprehensive analysis of energy migration in photosynthetic pigment–protein light-harvesting complexes (LHCs), it is difficult to imagine that there are still some aspects of either physical or chemical phenomena occurring in LHCs that have not been touched upon by the keen investigators of photosynthesis. LHCs are essential parts of the sunlight conversion machinery of any photosynthetic organism [1,2]. Along with a protein shell serving as a rigid skeleton, each LHC contains chlorophylls or bacteriochlorophylls, pheophytins, and carotenoids—the chromophores responsible for visible light absorption, energy transport, and charge separation [3]. The chromophore locations are rigidly fixed in LHCs, thus creating unique reciprocal orientations of pigments for each complex [4]. These orientations are not random; they determine a matrix of interaction energies between molecules that provides the characteristic paths of energy migration in the complex [5].

Like the drosophila fly, which was the key object of research on the nature of genetic mutations, LHCs of purple bacteria have become a classic example of the successful application of quantum theories from solid physics to simulate the linear and nonlinear optical response of pigment–protein complexes [4]. From the point of view of theoretical physics, LHCs of purple bacteria are ideal objects for studies [6]. In comparison with plant or cyanobacterial core complexes of photosystem I, peripheral complexes such as LHCI and LHCII, FMO complexes, or photosystem II, the main chromophores in LHC of purple bacteria, bacteriochlorophylls (BChl), form ring-like structures [2,4]. In general, the photosynthetic apparatus of purple bacteria contains two types of LHCs—the core and peripheral complexes (named LH1 and LH2, respectively). Both LH1 and LH2 consist of small one-helix α- and β-subunits that form heterodimers as the main building blocks of the LHCs and serve as binding sites for BChl molecules [7,8,9]. LH1 contains the most important pigment–protein complex of any photosynthetic species—the reaction center (RC). In addition to four BChl molecules, the RC includes two metal-free bacteriopheophytin molecules. Being absorbed by BChl molecules of LH2 and LH1, light quanta are transformed into excited states of the antenna and sequentially relax to the excited states of the RC, where the chemical reactions of charge separation occur [10,11,12,13].

**Figure 1:**
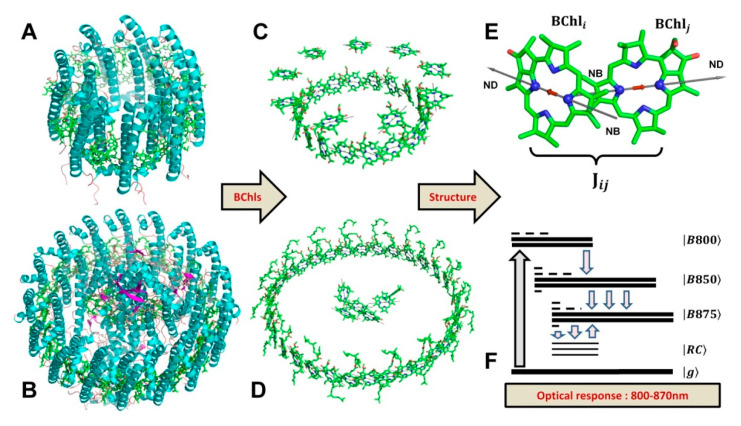
Crystal structures of LH2 from *Rhodopseudomonas acidophila* (**A**) and LH1-RC from *Thermochromatium tepidum* (**B**) illustrate typical molecular arrangements of peripheral (**A**) and core (**B**) light-harvesting complexes from purple bacteria. The protein framework of LH2 provides $C_{n}$ -type symmetry (in this case n=9) for BChl molecules in the complex (**C**), whereas the arrangement of BChl molecules in LH1 (**D**) is more ellipse-like, which corresponds to $C_{2}$ symmetry. The optical properties of LH1-RC and LH2 in the 800–870 nm region are entirely determined by the Q_y_ electronic excited state of BChl. The effective transition moment of this state is oriented roughly along the NB–ND direction of the porphyrin ring of BChl (**E**). The mutual orientation of BChl molecules in antennas and RC determines the interaction energies $J_{ij}$ between pigments, which, in turn, form an exciton manifold of antenna excited states and partially control the energy transport towards RC (**F**). X-ray data taken from the Protein Data Bank (https://www.rcsb.org/ accessed on 19 August 2021): 2fkw.pdb and 3wmm.pdb files.

LH2 complexes of different species of purple bacteria can be composed of seven [14], eight [8], or nine [9] helixes of α- and β-subunits. Since each subunit contains three BChl molecules, the total numbers of BChl molecules in LH2 are 21, 24, and 27 respectively. The optical properties of LH2 and LH1 in the 800–900-nm range are determined by the Q_y_ electronic transition of BChl. LH2 has two sharp peaks at 800 and 850 nm, belonging to the B800 and B850 rings of LH2, respectively. LH1 has only one ring of BChl molecules, leading to the appearance of only one absorption band at 875 nm for BChl a-containing species. Altogether, the BChl of LH2 and LH1 complexes form a manifold of excitation electronic states that direct the energy flow from LH2 to LH1 and, finally, to the RC (Figure 1). Interpretations of the spectral features of bacterial LHCs and RCs, as well as theories describing the energy transfer in antenna and their capture by RCs, have been changing quite dramatically over the past half-century [15,16,17,18,19,20,21].

For a fairly long time, the model of random walks of singlet excitations over a lattice of BChl monomers (or dimers) [22] remained the mainstream theoretical approach [23,24,25]. However, many steady-state absorption, time-resolved, and fluorescence spectroscopy data [26] and laser spectroscopy of ultra-high temporal resolution [27,28] contradicted this view of the nature of energy migration. The development of the energy transport theory based on the conception of delocalized exciton states in LHCs of purple bacteria [29] helped to predict the circular arrangement of pigments in LH2 and to explain many spectroscopy features of bacterial LHCs. The X-ray structural data that appeared later [8,9,30] proved the reality of ring aggregates of BChl molecules in LH2 and LH1 from purple bacteria. Since that time, the description of the processes of transfer and capture of excitation energy in bacterial photosynthesis on the basis of molecular exciton concepts has become generally accepted.

Absorption bands of LHCs usually contain dozens (in LH2, LH1), hundreds (in photosystem I [31,32], photosystem II), and thousands (in chlorosomes) of unresolved electronic lines, which could lead to ambiguous interpretations of photosynthetic processes if the proper theoretical approach would not be considered [1,33,34]. The electronic spectra of pigments are broadened by their nature due to interaction with the local environment and vibronic states belonging to electronic states [35,36,37]. Thus, it is not surprising that the modern studies on simulations of energy transport in LHCs are mostly focused on the problem of electron–phonon coupling effects [1,35,38]. Together with the electron–phonon coupling, the excitation energies of antenna pigments and the interaction energies between them are the key parameters [39] of any exciton theory that quantifies energy migration in LHCs (and are the subject of many debates). A small number of pigments responsible for absorption at 800–900 nm and the high symmetry of LH1 and LH2 made them the most suitable LHC systems to simulate numerous spectroscopy data using exciton–relaxation theories of different levels of sophistication: linear optics [40,41,42,43,44], pump–probe spectroscopy [45,46,47], single-molecule spectroscopy [48,49], coherence spectroscopy [50,51], and two-dimensional coherent electronic spectroscopy [52,53,54,55].

The objective of our work was to perform calculations with the classical Frenkel exciton Hamiltonian in order to demonstrate the effect of the location of BChl molecules and their mutual orientation in LH2 and LH1 on the spatial arrangement of vectors of the exciton transition moments $D_{\alpha }$. Generally, according to the classical theory, only $D_{\alpha }^{2}$ values are used to calculate the optical response of a system, and therefore, the directions and positions of $D_{\alpha }$ do not influence the exciton band intensity. We noticed that the transition moments of pigments in the exciton representation reflect, through the material Hamiltonian, the peculiarities of pigment locations in LHCs. Appling the visualization of $D_{\alpha }$ vectors calculated for the Q_y_ transitions of LH2 and LH1 complexes, we analyzed the structures of LHCs from purple bacteria in terms of the $D_{\alpha }$ spatial arrangement.

Primarily, we demonstrate how the exciton transition moments behave depending on BChl mutual orientation considering a system of two BChl molecules. The B850 ring is used as a model system and the effect of closing the pigments in the ring is shown. The following step was to visualize $D_{\alpha }$ vectors for the B800 ring and for LH2 and LH1 complexes. Finally, the outcomes of the visualization and possible interpretations of the results are discussed.

## 2. Theory

We adopted a Frenkel exciton Hamiltonian to model the optical response of a system of interacting BChl molecules. To demonstrate some peculiar properties of electronic transition moments of a system in the exciton representation, the dipole–dipole approximation was used to calculate the coupling energies between molecules. The extended dipole–dipole approximation is preferable for estimating the coupling more accurately [56]; however, the simplified point dipole model for interacting pigments will not restrict the generality of our results [39]. Thus, considering a system of N two-level pigments, a material Hamiltonian is written as

(1) $$H_{mat}=\hbar \underset{n}{\sum }\Omega_{n}B_{n}^{+}B_{n}+\frac{\hbar }{2}\underset{n\neq m}{\sum }J_{mn}\left(B_{m}^{+}B_{n}+B_{n}^{+}B_{m}\right)$$

where $\Omega_{n}$ is the energy of the Q_y_ electronic state of BChl. $J_{mn}$ is the coupling energy in the dipole–dipole approximation. If we denote the excited state of the *n*th BChl as |n〉 and the ground state as |0〉, then the boson creation and annihilation operators are $B_{n}^{+}=\left|n\rangle \langle 0\right|$ and $B_{n}=\left|0\rangle \langle n\right|$, and n=1,…, N, which obeys the following commutation rules: $\left[B_{n},B_{n}^{+}\right]=1$. In our study, we restricted ourselves to a pure electronic Hamiltonian and exploring its properties; we did not consider vibronic and electron–vibronic Hamiltonians. Such simplifications would be unacceptable when modeling optical spectra of a system; however, to reveal the relationship between the magnitude and direction of transition moments from the spatial arrangement of pigments, the Frenkel Hamiltonian was sufficient.

To transform the Hamiltonian (1) to the exciton representation, the eigenstates $c_{n}^{\mu }$ and eigenvalues $\epsilon_{\mu }$ had to be calculated by the diagonalization of (1). Then, the Hamiltonian (1) can be recast in the following form:

(2) $$H_{ext}=\hbar \underset{\mu }{\sum }\epsilon_{\mu }b_{\mu }^{+}b_{\mu }$$

where μ=1…N, $b_{\mu }^{+}=\underset{n}{\sum }c_{n}^{\mu }B_{n}^{+}$, and $b_{\mu }=\underset{n}{\sum }c_{n}^{\mu }B_{n}$ as the creation and annihilation operators in the exciton representation. $\epsilon_{\mu }$ is the energy of exciton states without taking into account the phonons and bath influence on the system.

A key parameter of any theory of optical response is the transition moment, which determines the intensity of a transition from the ground state to the electronic excited state of a pigment. The BChl molecule has four pronounced optical transitions in the visible region named B_x_, B_y_, and Q_x_, Q_y_. We performed all our simulations for the state with the lowest excitation energy, namely for the Q_y_ transition. The direction of the Q_y_ transition moment of BChl was roughly collinear to the NB–ND direction in the porphyrin ring. Assuming that $d_{i}$ is a vector of the effective Q_y_ moment of the *i*th BChl molecules, we could calculate the transition moments of the system of BChl molecules, $D_{\mu }$, in the exciton representation as follows:

(3) $$D_{\mu }=\underset{n}{\sum }c_{n}^{\mu }d_{n}$$

Any effective transition moment can be written as $d_{i}=R_{B}^{i}-R_{E}^{i}$, where $R_{B}^{i}$ and $R_{E}^{i}$ are position vectors of a transition moment for a certain state ($R_{B}^{i}$ and $R_{E}^{i}$ are the beginning and the end points). For a system of transition moments corresponding to the interacting pigments, $R_{B}^{i}$ and $R_{E}^{i}$ can be written as

(4) $$R_{B}^{i}=R_{C}+r_{B}^{i}$$

(5) $$R_{B}^{i}=R_{C}+r_{B}^{i}$$

where $R_{C}$ is the center of mass of the system. Then, the transition moment is $d_{i}=R_{B}^{i}-R_{E}^{i}=r_{B}^{i}-r_{E}^{i}$. $r_{B}^{i}$ and $r_{E}^{i}$ are vectors connecting the center mass and the beginning and the end points of transition moments. Thus, shifting the position of the system into the center of mass, the moments in the coordinate representation are $d_{i}=r_{B}^{i}-r_{E}^{i}$, and the following transition moments in the exciton representation are recast in the following form:

(6) $$D_{\mu }=\underset{n}{\sum }c_{n}^{\mu }d_{n}=\underset{n}{\sum }c_{n}^{\mu }r_{B}^{n}-\underset{n}{\sum }c_{n}^{\mu }r_{E}^{n}$$

The shift to the center of mass is an important procedure for studying exciton transition moments of a system since it allows for estimating the exciton effects associated with the location of the pigments in LHCs.

## 3. Results

### 3.1. Dimer of BChl Molecules

**Figure 2:**
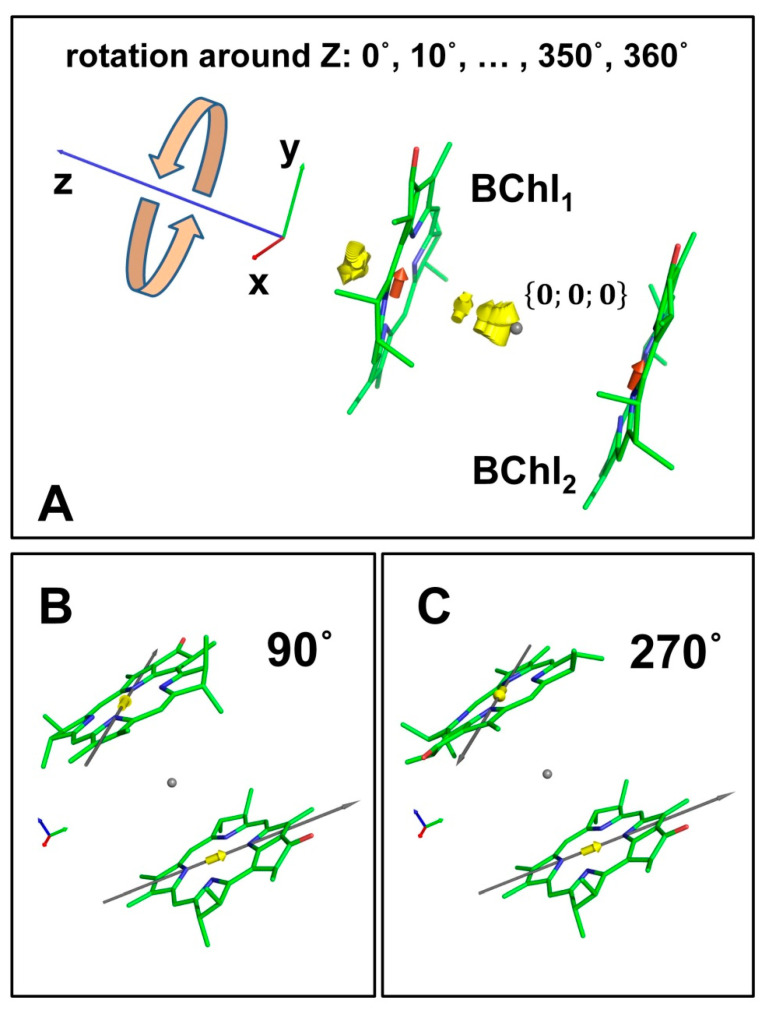
BChl molecule dimer transition moments in the exciton representation (yellow arrows) are shown in one plot (**A**) for each angle of *BChl*_2_ rotating around the z-axis. Red arrows are the Q_y_ transition moments of *BChl*_1_ and *BChl*_2_ in the coordinate representation. Two positions of BChl molecules at which the coupling energy is equal to zero are presented in separate plots (**B**,**C**). Gray points are the origin of coordinates.

The elementary model of interacting pigments is a dimer. Let us consider two pigment molecules, *BChl*_1_ and *BChl*_2_, located on the z-axis at a distance of 10 nm from each other (Figure 2). The molecules are oriented so that the vector of the NB–ND direction of each BChl molecule lies in the XY plane. Since we used the dipole–dipole approximation to estimate the coupling between pigments, the effective length of the Q_y_ transition moment was assumed to be 1.27Å [1]. Placing the dimer in the center of mass, we have the following coordinates for the end and beginning points of the transition moments in the coordinate representation:

(7) $$d_{BChl_{1}}=r_{B}^{BChl_{1}}-r_{E}^{BChl_{1}}={\left\{-0.02;-0.61;-5.00\right\}}_{B}^{BChl_{1}}-{\left\{-0.01;0.66;-5.00\right\}}_{E}^{BChl_{1}}$$

(8) $$d_{BChl_{2}}=r_{B}^{BChl_{2}}-r_{E}^{BChl_{2}}={\left\{-0.02;-0.61;5.00\right\}}_{B}^{BChl_{2}}-{\left\{-0.01;0.66;5.00\right\}}_{E}^{BChl_{2}}$$

These vectors, (7) and (8), were the initial positions before we began to rotate the *BChl*_2_ around the z-axis in 10-degree increments using the rotational matrix in Euclidian space.

For each position of BChl, the eigenstates and eigenvalues of the Hamiltonian (1) were calculated assuming the energies of pigments as $\Omega_{BChl_{1}}=12,500\;{\mathrm{cm}}^{-1}$ and $\Omega_{BChl_{2}}=12,550\;{\mathrm{cm}}^{-1}$. In fact, both the isoenergetic and non-isoenergetic models demonstrated the same effect (we did calculations for both), and thus, the results are presented only for the non-isoenergetic model. The analytical expressions for eigenstates and eigenvalues of the dimer model are well known [36], but we performed all the simulations numerically applying computational procedures that process systems containing any number of molecules.

**Figure 3:**
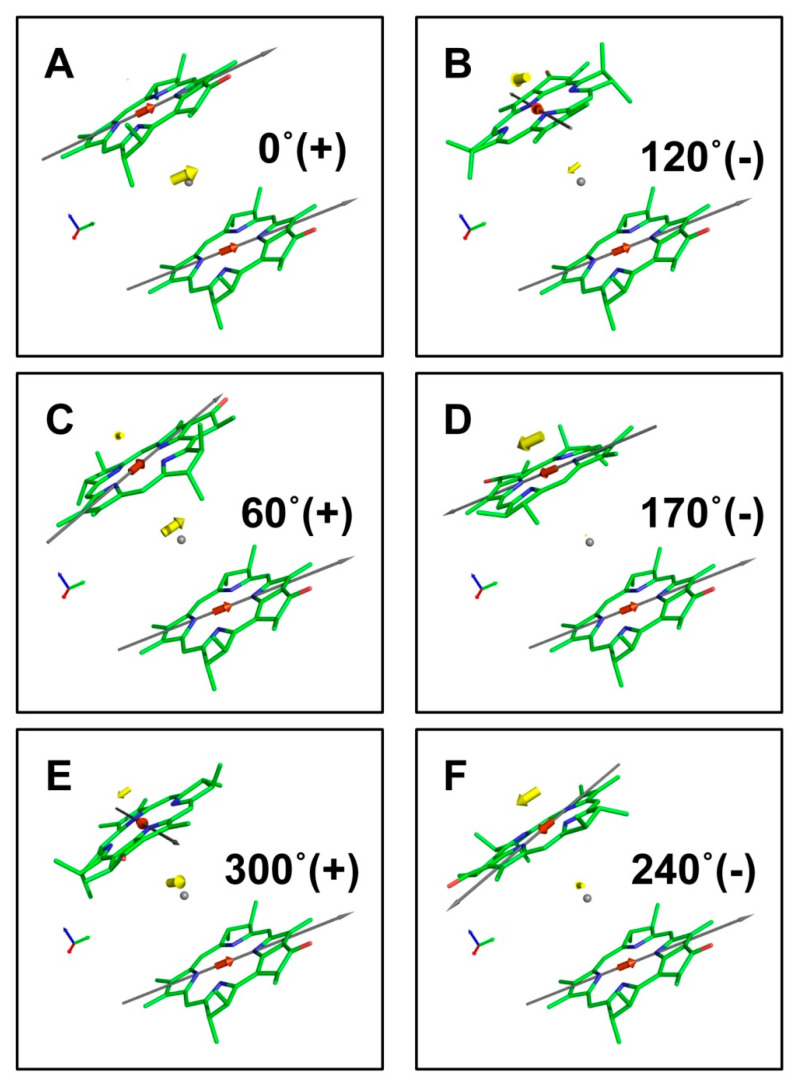
Q_y_ transition moments of BChl dimer in the coordinate (red arrows) and the exciton (yellow arrows) representations. Gray arrows indicate the NB–ND direction in the pigment molecule. The angle of rotation and the sign of coupling (+/–) are shown for each configuration of BChl molecules. Gray points are the origins of coordinates. Dimer configurations with positive coupling are shown in (**A**,**C**,**E**); negative coupling in (**B**,**D**,**F**).

The results of the modeling are shown in Figure 2 and Figure 3 and in Table A1 of Appendix A. Plot A in Figure 2 depicts molecules in the initial positions, and the red arrows are the transition moments $d_{i}$ in coordinate representation. The yellow arrows are a set of transition moments $D_{\mu }$ in the exciton representation calculated for angles, $\varphi_{k}=10k$, k=1,2,…,35, at which the *BChl*_2_ was rotated around the z-axis. Plots B and C show two special cases, when $J_{BChl_{1}BChl_{2}}\left(\varphi \right)=0$ and $d_{i}$ and $D_{\mu }$ coincide with each other.

Figure 3 demonstrates how the sign of coupling energy determines the spatial arrangement and intensities of $D_{\mu }$. If transition moments are repelled, the sign of the coupling energy $J_{nm}$ is positive. If they are attracted, the sign of $J_{nm}$ is negative. The full set of dimer model parameters, including the visualization of exciton transition moments, is available in electronic form (Video S1).

### 3.2. B850 Ring

The LH2 complex from *Rhodopseudomonas acidophila* contains nine α,β-polypeptides arranged in a ring structure. Each pair of polypeptides binds three BChl molecules, two of which belong to the B850 ring and one to the B800 ring (Figure 1). Instead of immediately analyzing the exciton properties of the LH2 complex, we started with the B850 ring.

Similar to the dimer model, the coordinates of the BChl molecules, taken from 2fkw.pdb file, were shifted to the center of mass. Only after that, $r_{B}^{i}$ and $r_{E}^{i}$, the beginning and end points of the Q_y_ transition moments were evaluated. It is generally accepted that the exciton model of the B850 ring is not isoenergetic and the transition energies of α,β-BChl are supposed to be different. However, for greater persuasiveness, we did calculations first for the isoenergetic and then for the non-isoenergetic models. In the first case, all $\Omega_{n}$ were taken as $12,200\;{\mathrm{cm}}^{-1}$, while in the second case, $\Omega_{n}^{\alpha }=12,125\;{\mathrm{cm}}^{-1}$ and $\Omega_{n}^{\beta }=12,275\;{\mathrm{cm}}^{-1}$. The matrix of the interaction energies in the B850 ring, $J_{mn}$, is given in Table A2 of Appendix A. The signs of the interaction energies alternate, starting with the positive for the nearest molecules, and thus, the strongest interaction in the B850 ring corresponds to repulsion. The transition moments in the exciton representation $D_{\mu }$ were calculated accordingly (6).

It must be stressed that inhomogeneous broadening was not taken into account in the simulations since it requires the average over random variations of $\Omega_{n}$ in the material Hamiltonian of a system (1). This procedure was not used in order to emphasize the effects associated with the spatial orientation of the pigment molecules.

**Figure 4:**
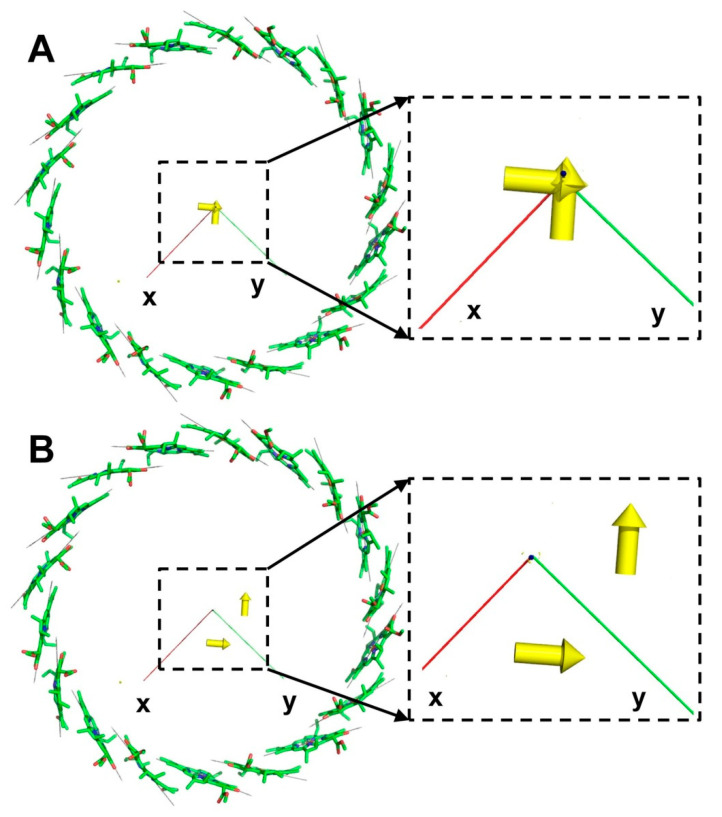
B850 exciton transition moments $D_{\mu }$ (yellow arrows) calculated for the isoenergetic (**A**) exciton Hamiltonian and for the Hamiltonian (**B**) with site energies, taken from [57].

The positioning of $D_{\mu }$ for the B850 ring is shown in Figure 4. It is known that due to the specific arrangement of BChl molecules in the B850 ring, the total dipole strength is mostly concentrated on two low exciton levels (frequently called ±k), while the dipole strength of the lowest level (k=0) is incomparably weaker. Considering the isoenergetic model of B850 (Figure 4A), we found very peculiar positions of $D_{\mu }$—two of the eighteen most intense transition moments were focused in the center of the ring and perpendicular to each other. When $\Omega_{n}^{\alpha }$ and $\Omega_{n}^{\beta }$ were different, the intense moments were slightly shifted from the center, but, nevertheless, they kept orthogonality (Figure 4B).

An intriguing simulation was a visualization of how Q_y_ transition moments in the exciton representation redistribute the dipole strength between each other and tend to be localized in the center of the ring. We did calculations starting with two BChl molecules, adding one molecule at a time, sequentially, until the B850 ring was closed. The full animation of $D_{\mu }$ localization for models containing BChl molecules from two to eighteen can be viewed by downloading Video S2.

### 3.3. LH2 and LH1 Complexes

Before proceeding with the simulation of $D_{\mu }$ for the LH2 complex, we first considered the B800 ring of LH2 as a separate system. The B800 ring consists of only nine BChl molecules. It is interesting that the spatial arrangement of these BChl molecules is significantly different from that of the B850 ring. According to the crystal structure [9], the distances between the nearest pigments in B800 and between the B800 and B850 rings are about 20Å. Such distances correspond to intermolecular interaction energies of the order of $20\;{\mathrm{cm}}^{-1}$, which is rather weak, and thus, BChl molecules of the B800 ring can be considered monomers. Nevertheless, expressions used in exciton theory are also valid for such interaction energies. Thus, we evaluated the material Hamiltonian (1) exactly as for the B850 ring, only taking into account $\Omega_{n}=12,500\;{\mathrm{cm}}^{-1}$. Unlike in the B850 ring, the signs of $J_{nm}$ in B800 are always negative. The matrix of the interaction energies, $J_{nm}$, is given in Table A3 of Appendix A.

**Figure 5:**
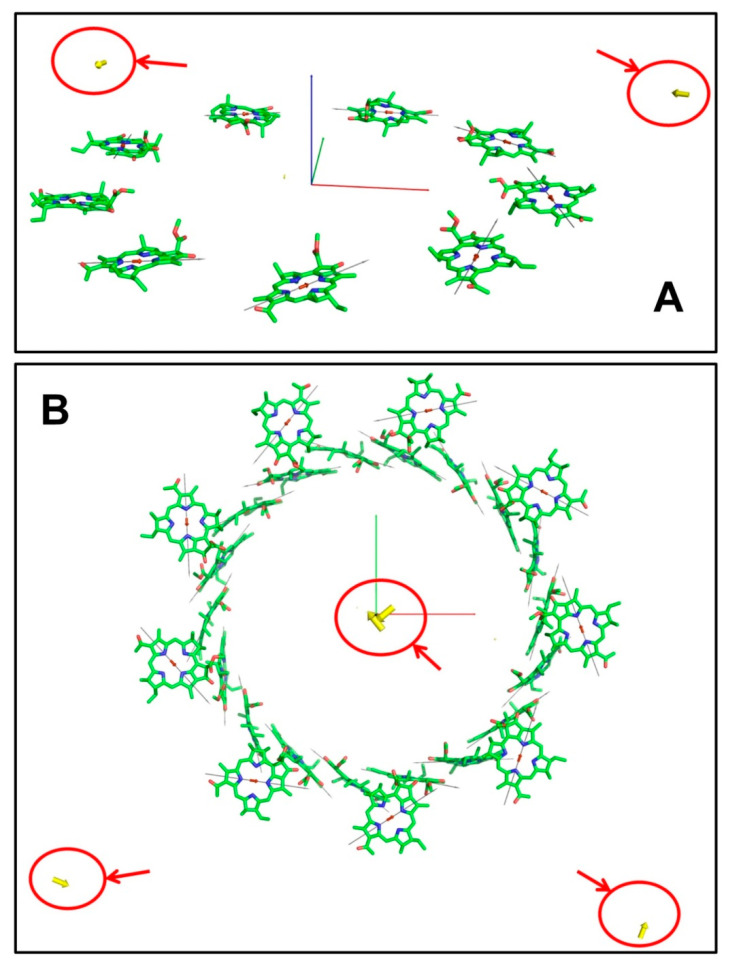
Visualization of the exciton Q_y_ transition moments $D_{\mu }$ (yellow arrows) for the B800 ring (**A**) and for the whole LH2 complex (**B**). Gray thin arrows are the NB–ND directions in BChl; small red arrows in the middle of pyrrole rings are the Q_y_ transitions in the coordinate representation. Red circles mark the locations of the transition moments for the two most intensive exciton states in the B800 ring and in LH2.

**Figure 6:**
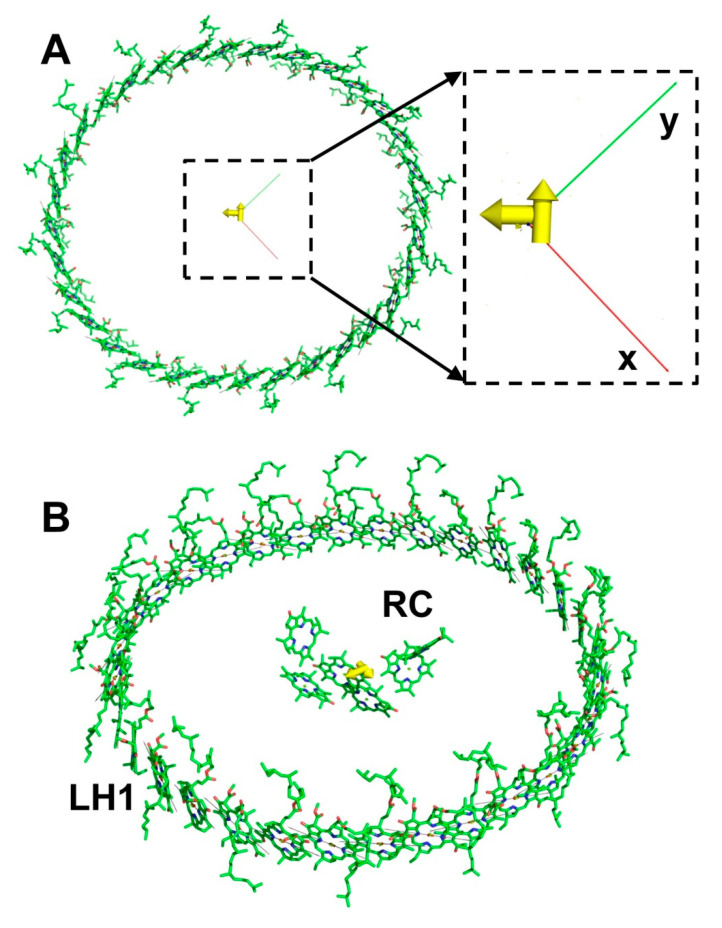
Exciton transition moments $D_{\mu }$ (yellow arrows) calculated for the core antenna of the LH1-RC complex in the case of the isoenergetic material Hamiltonian (**A**). The transition moments for the two most intensive exciton states are shown together with the pigments of RC (**B**).

The calculated $D_{\mu }$ vectors for the B800 ring are shown in Figure 5A. Comparing these results with those obtained for the B850 ring (Figure 4), it is clear that they are completely different. The distribution of the dipole strength over the exciton states in the B800 ring is similar to that in B850—the lowest state is weak, but the following two carry practically the entire dipole strength of the system, and the energies of these two intensive states are nearly the same. Their locations are marked with red circles in Figure 6A; the most intensive transition moments in the exciton representation are outside of the ring.

Collecting Hamiltonians together for the B800 and B850 rings provided the material Hamiltonian for the LH2 complex from *Rhodopseudomonas acidophila* as a 27-by-27 matrix. Since the rings of LH2 are spaced about 20Å apart, the eigenvalues of the LH2 Hamiltonian are those of B800 and B850, with slight alterations. The same goes for the $D_{\mu }$ spatial arrangement of LH2—the complex has two intensive transition moments in the center as well as two moments outside the rings (Figure 5B and Video S3).

The crystal structure of the core LH1-RC complex from *Thermochromatium tepidum* (3wmm.pdb) was used as a model to visualize the exciton Q_y_ transition moments of the core antenna of purple bacteria (Figure 1B,D). Before getting the transition moments in the coordinate representation, both the pigments of the antenna and the pigments of the RC were translated to the center of mass, and only then were the NB–ND directions of pigments read. The total number of pigments in the complex is 38—four BChl molecules and two bacteriopheophytin molecules belong to RC, and 32 BChl molecules are located in the antenna. The material Hamiltonian was created considering merely 32 BChl molecules of a core antenna with $\Omega_{n}=12,500\;{\mathrm{cm}}^{-1}$. Compared to LH2, pigments of the LH1 antenna were packed more densely, but they retained the same mutual orientation as in LH2. Thus, the coupling energies between the nearest molecules were slightly more than in LH2, while the alteration of signs coincided with LH2. The absolute values of $J_{mn}$ in LH1 varied from about $550\;{\mathrm{cm}}^{-1}$ to $0.5\;{\mathrm{cm}}^{-1}$. After diagonalization of the LH1 Hamiltonian, the dipole strength distribution of exciton states appeared to be the same as for LH2—the lowest state is very weak, and the two following states are most intensive; the contribution of other exciton states is negligible. The visualization of the LH1 exciton transition moments $D_{\mu }$ is presented in Figure 6A.

## 4. Discussion

In addition to the excitation energy, the transition moment is a fundamental characteristic of any excited state of pigments that are part of the LHC. Its squared value $D_{\mu }^{2}$, which determines the optical intensity of an exciton state, is a crucial quantity when relaxation rates, coherence effects, and the whole efficiency of energy transfer in the complex have to be assessed [33,34,58,59]. To the best of our knowledge, there are no reports describing a direct connection of $D_{\mu }$ with the spatial arrangement of pigments in LHC, and thus, here, we reveal certain key points of our approach regardless of other theoretical studies of LHC.

According to (3), transition moments in the exciton representation $D_{\mu }$ (in contrast to $D_{\mu }^{2}$) are not invariant with respect to the position of the complex in space. To eliminate this inconvenience, the system must be translated to the center of mass, $R_{C}$ in (4) and (5). Only in such a position can the vectors of effective excitation transition moments be considered characteristic parameters of the material Hamiltonian (1). As we have shown in the case of a dimer, the sign of the interaction energies $J_{mn}$ unambiguously determined the position of the most intensive transition in the dimer (Figure 3). When it was negative, the strongest dipole tended to be outside the pigment locations (Figure 3B,D,F). When it was positive, the strongest dipole tended to be located around the center of mass almost all the time (Figure 3A,C,E).

The same effect of the sign of $J_{mn}$ was observed for the B800 and B850 rings. The most interesting thing was that the strongest transition moments of the B850 ring were focused in the center of the ring and perpendicular to each other (Figure 4), but those of the B800 ring were spaced apart (Figure 5A). The ideal focusing of $D_{\mu }$ took place for the isoenergetic Hamiltonian. It was obvious that there is always inhomogeneous broadening in a real system, and the perfect configuration of $D_{\mu }$ is most likely broken. However, taking into account that $\Omega_{n}^{\alpha }$ and $\Omega_{n}^{\beta }$ were different, and borrowing these values from elsewhere [57] produced the arrangement depicted in Figure 4B. The vectors slightly moved from the center but remained perpendicular. Thus, we believe that the inhomogeneous broadening can disturb the ideal picture of exciton moments but does not destroy it completely.

In contrast to the B850 ring, which had a positive sign for the nearest coupling energy, all the couplings in the B800 ring were negative, which led to a corresponding picture of the transition moments in the exciton representation shown in Figure 5A. The strongest moments were outside of the ring and perpendicular to each other. The locations of $D_{\mu }$ from the center of the ring were about 45Å. Finally, the locations of exciton transition moments for the whole LH2 complex can be seen in Figure 6B—two strong dipoles are in the center of LH2 and two strong dipoles are outside of LH2.

From the point of energy transport within the complex and between complexes such as the configuration of $D_{\mu }$, vectors in LH2 can be interpreted as follows. Each LH2 complex, as a system of interacting pigments described by a single Hamiltonian, possesses two strong moments lying outside of itself. The excited states corresponding to these transition moments can be considered as the states that effectively participate in the inter-complex energy exchange since they can actively interact with transition moments of the other complexes, particularly with those located in the center of a complex. Consequently, any LH2 has exciton states that mostly preserve the energy in the system (exciton states of B850) and the states that are responsible for effective energy transport to the others (exciton states of B800). This interpretation was partially confirmed by the experimental measuring of inter- and intra-complex energy exchange rates in different species of purple bacteria [5].

Unlike LH2, BChl molecules of the LH1 core antenna formed an ellipse-like structure and had C_2_ symmetry; however, this feature did not affect the positions of the most intense exciton transition moments, just like in the B850 ring, at the center of LH1-RC complex, where the RC is located. In light of these results, it is clear why LH1-RC is similar to the B850 ring mutual orientation of BChl molecules in the core antenna—to focus the moments of the most intense exciton states around the location of the RC, thus providing effective energy transport from the antenna to the RC and decreasing the probability of reverse energy flow. It is worth mentioning that the moments of the lowest exciton energies of LH2 and LH1 were located approximately in the center mass, which can also be considered an advantage in the case of the LH1-RC complex.

Considering the fact that the material Hamiltonian of any system of interacting pigments can be optimized with respect to a desirable localization of transition moments $D_{\mu }$, it might be expected that $D_{\mu }$ vectors could be used as one of the key parameters of optimization, along with the optical spectra of a system. We are looking forward to applying this approach of transition moment visualization to compare LHCs of other photosynthetic species, and in particular, to evaluate different pathways of energy transport in them.

## 5. Conclusions

To summarize our results, we conclude that the specific mutual orientation of BChl molecules in LH1 and LH2 complexes from purple bacteria, as well as the high symmetry of those complexes create various spatial arrangements of the electronic transition moments, which can be used for the interpretation of the features of energy transport in the membranes of purple bacteria. To demonstrate the corresponding effects, all the simulations and visualizations were done for the Q_y_ electronic transition of BChl. We considered a standard Frenkel exciton Hamiltonian omitting the interactions with phonons and the local protein environment. Since we did not perform fitting of the experimental optical spectra, such simplification of the system Hamiltonian was acceptable for the purposes of our study. Based on the outcomes of earlier investigations, parameters such as the excitation energy and the transition moment of monomeric BChl were used to create the LH2 and LH1 exciton models and make visualizations of $D_{\mu }$. A key point of visualization is to translate a system of pigments to the center of mass before the calculation of transition moments. Without this procedure, $D_{\mu }$ are not invariant with respect to the position of the system in space. Thus, the visualization of transition moments for the B800 and the B850 rings from the LH2 complex revealed different pictures of $D_{\mu }$ vectors for these rings, despite the fact that the distributions of exciton intensities of B800 and B850 ($\sim D_{\mu }^{2}$) demonstrated distinct similarity. The strongest $D_{\mu }$ of B800 lie outside of the ring (outer transitions), while those of B850 are focused in the center of the ring (inner transitions). Finally, the visualization of $D_{\mu }$ for LH2 provided the following pattern: two strong transitions were outside of LH2 and the other two are perpendicular and at the center of LH2 (see Video S3). The core antenna of LH1-RC was characterized by the same as in the B850 location of the strongest $D_{\mu }$ in the center of the complex, which actually coincides with the RC. Further application of the generalized Förster theory [60] will make it possible to estimate the energy transfer rates between the peripheral antenna, core antenna, and the reaction centers.

## Data Availability

No additional data available.

## Supplementary Materials

The following are available online at https://www.mdpi.com/article/10.3390/ijms221810031/s1.

## Appendix A

**Table A1:** Parameters of the BChl dimer model: Z-coordinates of the *BChl*_1_ and *BChl*_2_ Q_y_ transition moments in the site representation are –5 Å and 5 Å. These moments lie on a line connecting the NB and ND nitrogen atoms of the pyrrole ring of BChl. $J_{12}$ is coupling energy between BChl molecules calculated in dipole–dipole approximation. $Z_{1}^{NB}$ and $Z_{2}^{NB}$ are the Z-coordinates of *BChl*_1_ and *BChl*_2_ Q_y_ transition moments in the exciton representation. $D_{1}^{2}$ and $D_{2}^{2}$ are the squared exciton transition moments. The parameters are calculated for different angles between transition moments in the site representation.

	0°	30°	60°	120°	170°	240°	300°	330°
$J_{12}$	188.41	163.14	94.18	−94.24	−185.53	−94.17	94.21	163.18
$Z_{1}^{NB}$	7.06	7.05	7.01	0.91	0.47	0.91	7.01	7.05
$Z_{2}^{NB}$	0.47	0.54	0.91	7.01	7.06	7.01	0.91	0.54
$D_{1}^{2}$	0.01	0.23	0.84	0.84	0.04	0.84	0.84	0.23
$D_{2}^{2}$	3.23	3.01	2.41	2.41	3.21	2.41	2.41	3.01

**Table A2:** Interaction energies between BChl molecules in the B850 ring, calculated in the dipole–dipole approximation. The Q_y_ transition moments are considered to be collinear to the NB–ND direction of the porphyrin ring of BChl. The crystal data were taken from 2fkw.pdb file.

	B601	C502	D602	E503	F603	G504	H604	I505	J605	K506	L606	M507	N607	O508	P608	R509	S609
A501	388.9	−53.7	14.0	−6.4	3.0	−2.0	1.2	−1.0	0.7	−1.1	0.9	−2.0	2.5	−6.6	12.5	−52.3	333.3
B601		360.5	−35.2	12.4	−4.1	2.6	−1.1	1.0	−0.4	0.8	−0.4	1.1	−1.0	3.1	−4.2	14.4	−35.8
C502			366.1	−53.1	14.4	−6.7	3.2	−2.0	1.2	−1.1	0.7	−1.0	1.0	−2.0	2.5	−6.6	12.3
D602				350.3	−35.2	12.6	−4.0	2.5	−0.9	0.9	−0.3	0.7	−0.3	1.1	−1.0	3.0	−3.8
E503					354.2	−52.0	13.9	−6.3	2.8	−1.9	1.0	−0.9	0.6	−1.0	0.9	−1.9	2.3
F603						381.7	−37.6	12.8	−4.1	2.5	−0.9	0.9	−0.4	0.7	−0.4	1.2	−1.0
G504							381.5	−54.9	14.7	−6.8	3.2	−2.0	1.2	−1.1	0.8	−1.1	1.0
H604								402.3	−39.7	13.7	−4.4	2.7	−1.2	1.1	−0.5	0.8	−0.5
I505									378.4	−53.7	14.1	−6.5	3.1	−2.0	1.2	−1.0	0.7
J605										340.9	−33.1	11.9	−3.9	2.4	−1.0	0.9	−0.4
K506											364.2	−53.2	14.5	−6.6	3.1	−2.0	1.2
L606												359.7	−36.2	12.4	−4.0	2.5	−0.9
M507													379.9	−53.5	14.2	−6.5	3.0
N607														355.2	−36.4	12.8	−4.0
O508															378.3	−54.9	14.4
P608																389.6	−36.6
R509																	362.4

**Table A3:** Interaction energies between BChl molecules in the B800 ring, calculated in the dipole–dipole approximation. The Q_y_ transition moments are considered to be collinear to the NB–ND direction of the porphyrin ring of BChl. The crystal data are taken from 2fkw.pdb file.

	C702	E703	G704	I705	K706	M707	O708	R709
A701	−24.9	−2.8	−0.8	−0.3	−0.3	−0.7	−2.7	−23.7
C702		−24.2	−2.9	−0.7	−0.3	−0.3	−0.7	−2.7
E703			−24.4	−2.8	−0.7	−0.3	−0.3	−0.7
G704				−25.5	−2.9	−0.7	−0.3	−0.3
I705					−24.2	−2.6	−0.7	−0.3
K706						−23.3	−2.6	−0.7
M707							−22.7	−2.6
O708								−23.4
